# Therapeutic Effects of Fenofibrate Nano-Emulsion Eye Drops on Retinal Vascular Leakage and Neovascularization

**DOI:** 10.3390/biology10121328

**Published:** 2021-12-15

**Authors:** Li Huang, Wentao Liang, Kelu Zhou, Ronald A. Wassel, Zachary D. Ridge, Jian-Xing Ma, Bing Wang

**Affiliations:** 1 Department of Ophthalmology, Fujian Medical University Union Hospital, 29 Xinquan Road, Gulou District, Fuzhou 350001, China; li-huang@ouhsc.edu; 2 Department of Physiology, University of Oklahoma Health Sciences Center, Oklahoma City, OK 73104, USA; wentao-liang@ouhsc.edu (W.L.); Kelu-zhou@ouhsc.edu (K.Z.); 3 EyeCro, LLC, Oklahoma City, OK 73104, USA; dwassel@eyecro.com (R.A.W.); Zridge@eyecro.com (Z.D.R.)

**Keywords:** fenofibrate, nanomedicine, eye drop, age-related macular degeneration, diabetic retinopathy, PPARα, vascular leakage

## Abstract

**Simple Summary:**

Age-related macular degeneration (AMD) and diabetic retinopathy (DR) are the leading causes of blindness and lack non-invasive drug treatments. Macular edema and neovascularization are the key pathogenic features responsible for vision loss in AMD and DR. Clinical studies showed that fenofibrate, a PPARα agonist, has robust therapeutic effects on macular edema and retinal neovascularization after oral administration. However, the efficacy of oral fenofibrate remains to be improved, due to limited amounts of the drug delivered to the retina after systemic administration. To increase the drug availability in the retina, this study developed a novel nano-emulsion fenofibrate eye drop. Topical administration of the fenofibrate eye drop delivered significantly higher drug concentrations to the retina/vitreous, relative to the systemic administration. The fenofibrate eye drop alleviated retinal inflammation and vascular leakage in both DR and AMD models. Further, the fenofibrate eye drop also alleviated laser-induced choroidal neovascularization. These findings suggest that topical administration of the nano-emulsion-based eye drop of a PPARα agonist has potential to become a non-invasive therapeutic strategy for long-term treatment of DR and AMD.

**Abstract:**

Macular edema caused by retinal vascular leakage and ocular neovascularization are the leading causes of severe vision loss in diabetic retinopathy (DR) and age-related macular degeneration (AMD) patients. Oral administration of fenofibrate, a PPARα agonist, has shown therapeutic effects on macular edema and retinal neovascularization in diabetic patients. To improve the drug delivery to the retina and its efficacy, we have developed a nano-emulsion-based fenofibrate eye drop formulation that delivered significantly higher amounts of the drug to the retina compared to the systemic administration, as measured by liquid chromatography–mass spectrometer (LC-MS). The fenofibrate eye drop decreased leukocytes adherent to retinal vasculature and attenuated overexpression of multiple inflammatory factors in the retina of very low-density lipoprotein receptor knockout (*Vldlr^−/−^*) mice, a model manifesting AMD phenotypes, and streptozotocin-induced diabetic rats. The fenofibrate eye drop also reduced retinal vascular leakage in these models. The laser-induced choroidal neovascularization was also alleviated by the fenofibrate eye drop. There were no detectable ocular toxicities associated with the fenofibrate eye drop treatment. These findings suggest that fenofibrate can be delivered efficiently to the retina through topical administration of the nano-emulsion eye drop, which has therapeutic potential for macular edema and neovascularization.

## 1. Introduction

The blood–retinal barrier (BRB) breakdown or vascular leakage can result in macular edema, which is the leading cause of vision loss in age-related macular degeneration (AMD) and diabetic retinopathy (DR) [1,2]. In neovascular AMD, a subretinal neovascular disease, new vessels grow from the choroid into the avascular outer retina and subretinal space. In proliferative DR, pathological neovascularization (NV) originates from retinal vessels. These pathological NV can result in vision loss. As such, amelioration of retinal vascular leakage and NV represents a major goal for the treatment of AMD and DR [2,3].

The key pathophysiological process in macular edema in DR and AMD is believed to be the BRB breakdown. The macromolecules in the plasma leak out of the retinal capillaries, resulting in retina edema, which most commonly occurs in the macula [4]. The breakdown of the BRB is often accompanied by inflammatory responses, involving various inflammatory factors, such as intercellular adhesion molecule-1 (ICAM-1) and vascular endothelial growth factor (VEGF) [5]. It has been shown that leukostasis, leukocyte adherence to the retinal vasculature, is increased in the retina of both AMD and DR models. The consequent impairment of the capillary endothelium leads to increases of vascular permeability and macular edema [6,7]. According to AMD Preferred Practice Pattern (PPP) guidelines, intravitreal injection of anti-VEGF agents is the most effective way to manage neovascular AMD and represents the first line of treatment [8]. For proliferative DR or macular edema, the anti-VEGF therapy is also considered a stand-alone treatment or in combination with panretinal photocoagulation [9]. However, a study argued that VEGF inhibitors are symptomatically effective in only 30% of patients with neovascular AMD in preventing the progression of neovascular AMD with some vision recovery [10,11]. Additionally, repetitive intravitreal injections have risks of causing side effects such as cataracts, bleeding, retina damage, and in severe cases, postinjection endophthalmitis. Furthermore, emerging evidence suggests that the anti-VEGF treatment may lead to exacerbation of vasoproliferative disease after cessation of initial anti-VEGF therapy [12]. Therefore, there is an unmet clinical need for a non-invasive, affordable, and long-term treatment for DR and AMD.

Fenofibrate, a FDA approved fenofibric acid derivative drug, is widely used to reduce triglycerides and total cholesterol while increasing high-density lipoprotein cholesterol in adults [13]. After absorption, fenofibrate is rapidly metabolized in tissues and plasma to its active metabolite, fenofibric acid, which is an agonist of peroxisome proliferator–activated receptor-α (PPARα) [14]. In addition to its lipid-lowering effects, fenofibrate is known to regulate other pathways involved in inflammation, angiogenesis, and cell survival [15,16,17]. The Fenofibrate Intervention and Event Lowering in Diabetes (FIELD) study reported that fenofibrate oral administration (200 mg/day) reduced the need for laser treatment for DR and reduced DME in people with type 2 diabetes [18]. This finding was further confirmed by an independent clinical study, the Action to Control Cardiovascular Risk in Diabetes (ACCORD)-Lipid trial, which provided evidence that fenofibrate added to simvastatin therapy in people with type 2 diabetes slowed down the progression of DR [19]. Our recent study has demonstrated that fenofibrate also reduced vascular leakage and downregulated the expression of inflammatory factors in neovascular AMD models [20,21].

Due to fenofibrate’s low aqueous solubility, it is difficult to formulate into a classic eye drop. Hence, this study aimed to develop a nano-emulsion-based fenofibrate eye drop that can deliver significant amounts into the retina. We also explored the therapeutic potential of the fenofibrate eye drop as a future convenient and non-invasive approach to reducing retinal vascular leakage and retinal inflammation in neovascular AMD and DR.

## 2. Materials and Methods

### 2.1. Preparation and Therapeutic Regimen of Fenofibrate Eye Drops

The fenofibrate nano-emulsion eye drops were prepared to treat animal models. The ingredients in the eye drops contained fenofibrate (Sigma-Aldrich, St. Louis, MO, USA), polysorbate 20 (Millipore Co., Bellerica, MA, USA), polysorbate 80 (Nanjing Well Chemical Co., Ltd, Nanjing, China), and isopropyl myristate (Millipore Co., Bellerica, MA, USA) and distilled water. According to the percentage of weight, 100 mL 0.1% fenofibrate eye drop included 0.1 g fenofibrate, 3.6 g isopropyl myristate, 22 g polysorbate 20, 22 g polysorbate 80, and 52.3 g distilled water. The reagents were mixed and stirred overnight. The viscosity index was measured to be 314.8. The average size of nanoparticles of fenofibrate was determined to be 2.192 nm with Malvern Nanosizer (Malvern Panalytical Ltd, Westborough, MA, USA) in Charlesson, LLC, Oklahoma City, OK, USA.

For therapeutic effects, experimental animals were randomized into two groups: (a) a group that received vehicle eye drop and (b) a group that received 0.1% fenofibrate eye drop for mice and 0.5% fenofibrate eye drop for rats. All treated groups received topical eye drops on the same daily regimen (dose volume for mice: 10 μL/eye; dose volume for rats: 20 μL/eye); bilaterally twice a day for 7 days. After applying eye drops, animals were held for at least 1 min to prevent them from claw scratching.

### 2.2. Animals

#### 2.2.1. Very Low-Density Lipoprotein Receptor Knockout (*Vldlr^−/−^*) Mice

Breeding pairs of *Vldlr^−/−^* mice in the C57BL/6J background were originally purchased from Jackson Laboratories. The breeding colony was maintained in standardized conditions. Male *Vldlr^−/−^* mice (28 days) were used in the experiments.

#### 2.2.2. Laser-Induced CNV

CNV was induced by laser photocoagulation in male wild-type (WT) C57BL/6J mice (10 weeks old; Jackson Laboratories, Bar Harbor, ME, USA). After anesthesia, the fundus was viewed with an imaging camera, and laser photocoagulation was induced using the image-guided laser system (Micron IV, Phoenix Research Laboratories, Pleasanton, CA, USA). Four laser burns at equal distance of approximately 2 optic disc diameters from the optic nerve were applied to each eye by a green argon laser pulse with a wavelength of 532 nm, a spot size of 50 μm, a duration of 100 ms, and a power level of 260 mW. Production of a cavitation bubble at the laser point was considered a sign of disruption of Bruch’s membrane, which leads to the formation of CNV. Therefore, only burns with bubbles were included in this study [22].

#### 2.2.3. Streptozotocin (STZ)-Induced Diabetic Rats

Rats were used for induction of diabetes as described previously [21]. Briefly, male Brown Norway (BN) rats (8 weeks old; Charles River Laboratories International, Inc., Wilmington, MA, USA) received a single intraperitoneal injection of STZ (Sigma-Aldrich Corp, St. Louis, MO, USA) with the dosage of 50 mg/kg body weight dissolved in 10 mM of citrate buffer (pH 4.5) after overnight fasting. The blood glucose was measured with a glucose analyzer (Beckman Instruments, Zürich, Switzerland), 3 days after the STZ injection and weekly thereafter. Only the animals with blood glucose higher than 350 mg/dL were used as diabetic models in this study. At 3 weeks after STZ injection, animals were treated with fenofibrate eye drop or vehicle eye drop twice daily for 7 days [23].

In all procedures, rodents were anesthetized with an intraperitoneal injection of a mixture of ketamine (50 mg/kg, Zetamine, MWI, Boise, ID, USA) and xylazine (5 mg/kg, AnaSed LA, MWI, Boise, ID, USA), and pupils were dilated with topical administration of 1% tropicamide ophthalmic solution (Bausch + Lomb, a division of Bausch Health US, LLC, Bridgewater, NJ, USA).

### 2.3. Tissue Distribution of the Drug

For tissue distribution study, C57BL/6J mice received 0.1% fenofibrate eye drops or vehicle eye drops at a dose volume of 10 μL/eye in both eyes. BN rats received 0.5% fenofibrate eye drops or vehicle eye drops at a dose volume of 20 μL/eye in both eyes. In comparison, the systemic treatment group of animals received intraperitoneal injections of fenofibrate with a dose of 2 mg/kg, once a day for 7 days in animals. Contents of fenofibrate and fenofibric acid in various animal tissues were measured by LC-MS 6 h after the final dose of fenofibrate.

Analysis was performed using an AB Sciex 4000 liquid chromatography–mass spectrometer (LC-MS) system (Atlantic Lab Equipment, Inc., Salem, MA, USA). It was coupled to a Nexera X2 ultra high-performance liquid chromatograph system (Shimadzu Corporation, Columbia, MD, USA), which delivers high-performance quantitation and identification across a wide range of LC flow rates. The analytical column was an Accucore C18 column 2.6 μm (50 mm × 2.1 mm) (Thermo Fisher Scientific Inc., Waltham, MA, USA). Mobile Phase A consisted of 0.1% formic acid dissolved in deionized water, and Mobile Phase B consisted of 0.1% formic acid dissolved in methanol. The analysis was performed using a simple isocratic mobile phase consisting of 80% phase B. The flow rate was 300 µL per minute, and the column temperature was maintained at 40 °C. The MS detector was operated in MRM mode at unit mass resolution with a dwell time of 100 ms for all test compounds. The optimized mass spectrometric parameters, MRM transitions for fenofibrate, and fenofibric acid were *m/z* 361.0 to 121 in positive ESI mode and *m/z* 319.0 to 232.9 in negative ESI mode, respectively. The ion source parameters were Curtain Gas (CUR) 20, Turbo Gas 1 (GS1) 30, Turbo Gas 2 (GS2) 60, Temperature (TEM) 400 °C, Entrance Potential (EP) 10, and Cell Exit Potential (CXP) 6 for maximum sensitivity [24].

### 2.4. Fundus Fluorescein Angiography (FFA)

FFA was performed with the retinal imaging microscope (Micron IV, Phoenix Research Laboratories, Pleasanton, CA, USA) at 5 and 10 min after 5% sodium fluorescein (100 μL/mouse) intraperitoneally injection. The difference in integrated intensity between 5 and 10 min was recorded as an indicator of vascular leakage as described previously [22]. The lesion areas were measured using ImageJ (National Institutes of Health, Bethesda, MD, USA). For *Vldlr^−/−^* mice, the numbers of fluorescein leakage spots at 5 min after injection were used for analysis.

### 2.5. Optical Coherence Tomography (OCT)

OCT (Envisu^TM^ R-series SDOIS system, Bioptigen, Inc., Morrisville, NC, USA) imaging was performed after animals were anesthetized with pupils dilated. The corneal and retinal thickness were measured at 500 µm intervals temporal and nasal to the optic nerve head in all images obtained from both horizontal and vertical scans, using the system software (InVivoVU, Bioptigen, Inc.), and averaged [25]. For laser-induced CNV mice, the CNV volume was quantified using ImageJ based on the volume formula as described [20,26].

### 2.6. RPE/Choroidal Flat-Mount Preparation and CNV Area Quantification

For laser-induced CNV mice, eyes were enucleated and fixed with 4% paraformaldehyde solution for 2 h. The CNV lesions were stained with Isolection B4 (IB4, 10 μg/mL, Life Technologies, New York, NY, USA) as described previously [22]. Fluorescence images were taken with Cytation 1 Cell Imaging Multi-Mode Reader (BioTek, Winooski, VT, USA), and the staining areas of CNV lesions were calculated using ImageJ.

### 2.7. Retinal Vascular Permeability Assay in Vldlr^−/−^ Mice and STZ-Induced Diabetic Rats:

Anesthetized animals were injected with Evans blue (30 mg/mL) (Sigma-Aldrich, St. Louis, MO, USA) over 10 s through the femoral vein using a glass capillary under microscopic inspection at the dosage of 1 μL/g body weight after anesthesia, as described previously [27]. The animals were placed on a warm pad to allow the circulation of Evans blue for 2 h. Then, the animals were perfused via the left ventricle with 1% paraformaldehyde in a citrate buffer (pH 4.2). Immediately after the perfusion, the eyeballs were enucleated, and the retinas were carefully dissected. Each retina was placed in an ultracentrifuge tube containing 200 μL formamide (Sigma-Aldrich, St. Louis, MO, USA), then incubated for 18 h at 70 °C. After incubation, the extract was ultracentrifuged (Beckman Coulter Optima TLX Ultracentrifuge, Indianapolis, IN, USA) at 70,000 rpm for 30 min at 4 °C. The supernatant was taken into a disposal cuvette. Absorbance was measured at 620 nm with a spectrophotometer (Beckman Coulter DU 800, Indianapolis, IN, USA). PBS was added into each ultracentrifuge tube, which had 100 μL left. The sample was homogenized and then centrifuged at 13,500 rpm for 20 min at 4 °C. The supernatant was taken into a disposal cuvette containing 900 μL Coomassie brilliant blue reagent (ThermoFisher, Waltham, MA, USA). Then, the whole mix was vortexed for 1 min, and absorbance was measured at 595 nm with the spectrophotometer. After calculating the concentration of protein from a standard curve, the concentrations of Evans blue dye in the retinal extracts were normalized by total retinal protein concentration.

### 2.8. Retinal Leukostasis Assay

Immediately after euthanasia, animals were perfused with 500 mL/kg of pre-warmed PBS to remove circulating blood cells and nonadherent leukocytes from the retinal vasculature. Then, the animals were perfused with fluorescein isothiocyanate-conjugated concanavalin A (FITC-ConA) (20 μg/mL in PBS; total volume of 250 mL/kg body weight; Vector Laboratory Inc., Burlingame, CA, USA) to label adherent leukocytes. An additional perfusion with 250 mL/kg of PBS was used to wash out the excess concanavalin A in the vasculature [28,29]. The enucleated eyeballs were fixed in 4% paraformaldehyde solution for 2 hs, and then, the retinas were flat-mounted in the anti-fading mounting media (Vector Laboratory Inc., Burlingame, CA, USA). Fluorescence images of FITC-labeled leukocytes in flat-mounted retinas were captured using Cytation 1 Cell Imaging Multi-Mode Reader. Numbers of adherent leukocytes in the main vessels and its primary vessel branch per retina were counted in a double-blind manner and compared among groups.

### 2.9. ELISA for VEGF, ICAM-1, and TNF-α

Protein levels of VEGF, ICAM-1, and TNF- α were individually measured in homogenates of the RPE/choroid complex of laser-induced CNV mice and retina of STZ-induced diabetic rats using DuoSet ELISA Development kits (R&D Systems Inc., Minneapolis, MN, USA) according to the manufacturer’s protocol.

### 2.10. Western Blot Analysis for PPARα:

Western blot analysis was performed as described previously [30]. Eyecups without the retina of laser-induced CNV mice and the retina of STZ-induced diabetic rats were dissected and homogenized. Protein concentrations in the homogenates were measured using the Bradford assay. The equal amount (50 μg) of total protein from each sample was resolved for analysis. Primary antibody dilutions were 1:1000 for the rabbit anti-PPARα antibody (600–636; Novus) and 1:5000 for a mouse anti–β-actin antibody (A5441; Sigma-Aldrich, St. Louis, MO, USA). The secondary antibody dilution was 1: 2000 for the horseradish peroxidase-labeled secondary antibody (1:2000 dilution, Santa Cruz Biotechnology, Dallas, TX, USA). Densitometry was performed using the ImageJ software and normalized by β-actin levels.

### 2.11. Electroretinography (ERG) Recording

ERG was recorded using the Espion Visual Electrophysiology System (Diagnosys, Lowell, MA, USA). Rats were dark-adapted for 16 h for scotopic ERG. The full ERG responses of both eyes were simultaneously recorded at the flash intensity of 600 cd·s/m^2^, and the data in both eyes were recorded, and the average of the left and right eyes were used for the analysis [31].

### 2.12. Intra-Ocular Pressure (IOP) Measurement

Icare tonometer (Icare Finland Oy, Vantaa, Finland) was used for the measurement of IOP. The animals were under anesthesia. No topical anesthetic is needed. The probe was made a gentle and brief contact with the eye while taking the measurement. The values were averaged in three independent measurements.

### 2.13. Histology

The eyeballs of animals were immersed for 24 h in Davidson fixative (35% distilled water, 20% formaldehyde (4%), 10% glacial acetic acid, and 35% absolute ethanol). The eyeball was washed with PBS, followed by gradient ethanol dehydration. The eyeball was then transferred to 70% ethyl alcohol, embedded in paraffin, and 5 μm thickness sections were cut [32]. The slides were also stained with hematoxylin and eosin according to the procedure. Photographs were taken under Olympus Microscope (BX43, Olympus, Tokyo, Japan) attached to a digital camera (U-TV0.5XC-3, Olympus, Tokyo, Japan). For all eyes, retinal images were obtained at the same distance (1500 µm) from the optic nerve.

### 2.14. Statistical Analysis

The results were analyzed using GraphPad Prism 8 (GraphPad Software, Inc., La Jolla, CA, USA). Data were expressed as mean ± standard error (SEM). Quantitative data were analyzed and compared using an unpaired t-test. For data sets with two or more groups, statistical analyses were performed with analysis of variance (ANOVA) following a Tukey’s multiple comparisons test. *p* value < 0.05 indicates statistical significance.

## 3. Results

### 3.1. Tissue Distribution Studies

**Figure 1:**
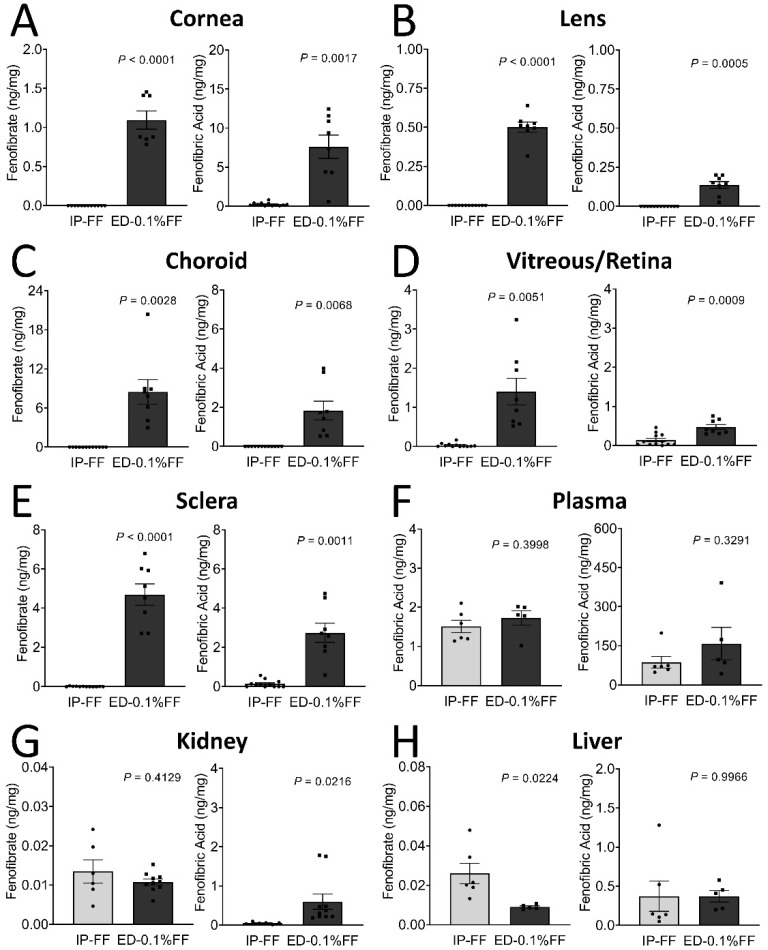
The tissue distribution of fenofibrate (FF) and fenofibric acid in mice following fenofibrate intraperitoneal injection (IP) and topical application of eye drops (ED). The fenofibrate and fenofibric acid levels were determined using LC-MS in tissues as indicated. (**A**–**H**) Left graph: The tissue distribution of fenofibrate. Right graph: The tissue distribution of fenofibric acid. The data were presented as mean ± SEM, analyzed by unpaired Student’s *t* test, *n* = 5–12 eyes or organs in each group.

The tissue distributions of fenofibrate and fenofibric acid in tissues of mice after the eye drop treatment are illustrated in Table 1 and Figure 1. Six hours following the last topical administration, fenofibrate and fenofibric acid, the active form of fenofibrate, were measured in six ocular tissues (cornea, lens, vitreous/retina, choroid, and sclera), two organs (kidney and liver), and plasma. As quantified by mass spectrometry, fenofibrate and fenofibric acid concentrations in the vitreous/retina were 0.033 ± 0.014 and 0.140 ± 0.044 ng/mg of total protein in the intraperitoneal injection group and 1.402 ± 0.341 and 0.472 ± 0.064 ng/mg in the fenofibrate eye drop group, demonstrating significantly higher drug delivery to the retina by the eye drop compared to the systemic administration (Figure 1D). Meanwhile, in all ocular tissues, the concentrations of fenofibrate and fenofibric acid in the eye drop group were significantly higher than in the intraperitoneal injection group (Figure 1A–E). Fenofibrate abundance was higher in the choroid followed by sclera, cornea, plasma, vitreous/retina, lens, kidney, and the liver. For fenofibric acid, however, the highest amount was observed in the plasma, followed by the cornea, sclera, choroid, and vitreous/retina. Despite the higher concentrations of the drug in ocular tissues by the fenofibrate eye drop; however, there was no statistically significant difference of the drug concentrations in plasma between these two administration routes (Figure 1).

### 3.2. Fenofibrate Eye Drop Alleviated Retinal Vascular Leakage in Vldlr^−/−^ Mice

**Figure 2:**
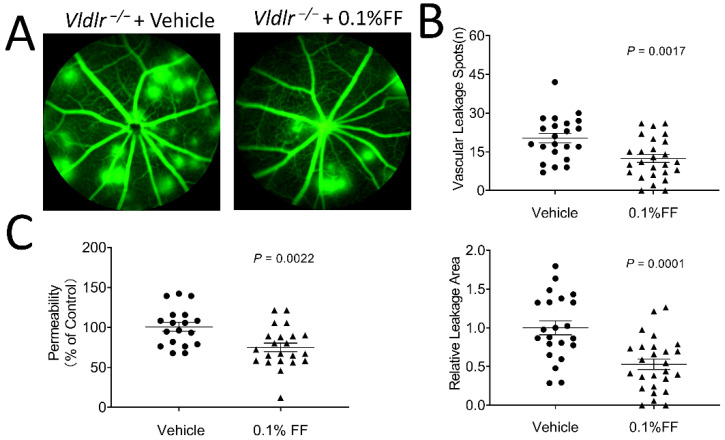
Fenofibrate eye drops alleviate fundus vascular leakage and permeability in *Vldlr*^−/−^ mice. The animals received 0.1% fenofibrate (FF) eye drop treatment (twice a day) for 7 days. Fundus fluorescein angiography was performed, and retinal vascular permeability was quantitated using Evans blue as a tracer. (**A**) Representative images showed the fundus vascular leakage (hyperfluorescent spots) corresponding to the areas of increased choroidal and retinal permeability. (**B**) The vascular leakage was quantified by the number of hyperfluorescent spots and the percentage of hyperfluorescent area in total retinal area in the 0.1% FF group (*n* = 26 eyes in each group) and the vehicle group (*n* = 22 eyes in each group). (**C**) The permeability of the retinal vasculature was measured in the 0.1% FF eye drop group (*n* = 22 eyes in each group) and vehicle group (*n* = 18 eyes in each group) using Evans blue dye as a tracer. The extracted Evans blue dye was normalized to the total retina protein. Data were expressed as mean ± SEM, analyzed by an unpaired Student’s *t* test.

To determine whether the fenofibrate eye drop reduced vascular leakage in *Vldlr*^−/−^ mice, the eye drops were administrated twice a day for 7 days from P28. As shown by FFA, the eyes administrated with the vehicle eye drop developed multiple leakage spots and large vascular leakage areas. In contrast, the mice with the fenofibrate eye drop treatment showed alleviated vascular leakage (Figure 2A,B). In addition, retinal vascular permeability was quantified using Evans blue permeability assay, and the result showed that retinal permeability was reduced by 25.73% in the fenofibrate eye drop group compared with the vehicle group (Figure 2C).

### 3.3. Fenofibrate Eye Drop Inhibited Leukocyte Adhesion to Retinal Vasculature in Vldlr^−/−^ Mice

**Figure 3:**
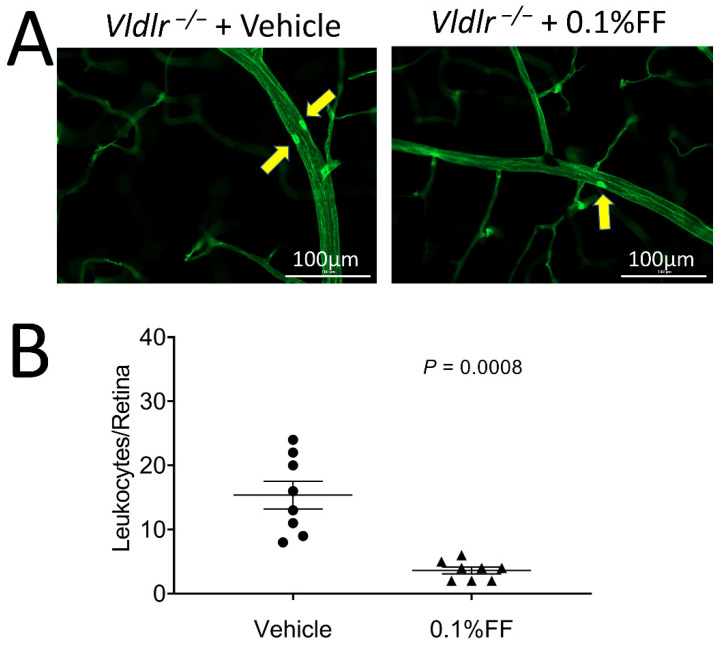
Fenofibrate eye drops inhibited the leukocyte adhesion in retinal vasculature of *Vldlr*^−/−^ mice. (**A**) Representative images of retinal leukostasis assay in *Vldlr*^−/−^ mice treated with vehicle (*n* = 8 animals per group) and 0.1% fenofibrate (FF) eye drops (*n* = 8 animals in each group). The arrows indicate the adherent leukocytes in retinal vessels. Scale bar: 100 μm. (**B**) Adherent leukocytes were quantified in flat-mounted retinas. Data were expressed as mean ± SEM, analyzed by an unpaired Student’s *t* test.

To evaluate the effect of the fenofibrate eye drop on suppressing retinal inflammation in *Vldlr^−/−^* mice, leukocytes adherent to the retinal vasculature were viewed and quantified following FITC-ConA perfusion. Fewer adherent leukocytes were observed in the retinal vasculature in *Vldlr^−/−^* mice treated with fenofibrate eye drop compared with the vehicle group (Figure 3A,B), suggesting that the fenofibrate eye drop alleviates retinal leukostasis in a neovascular AMD model.

### 3.4. Fenofibrate Eye Drop Suppressed Laser-Induced CNV

**Figure 4:**
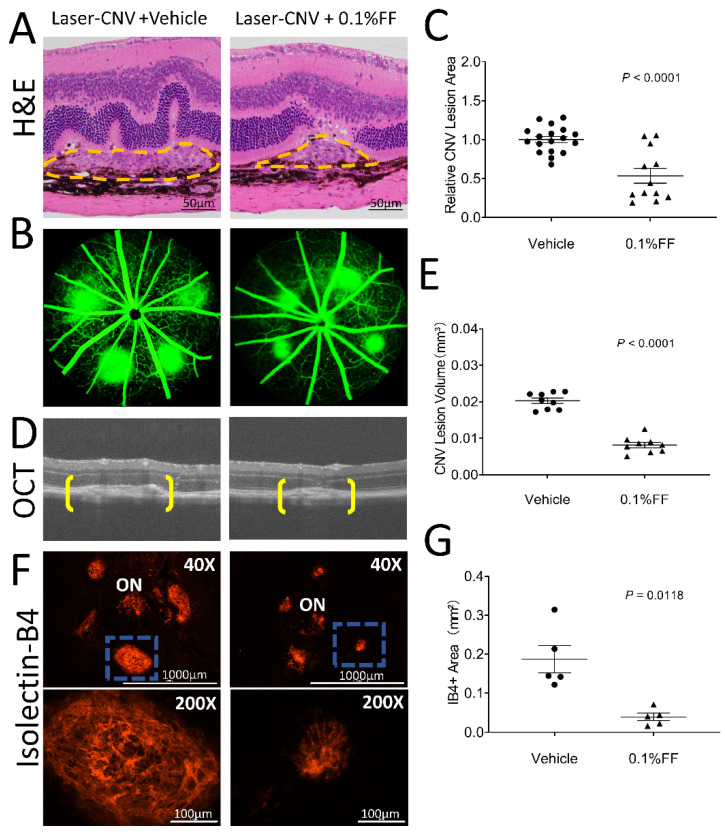
Fenofibrate eye drops suppressed laser-induced CNV. Laser-induced CNV was examined after the fenofibrate eye drop (FF) treatment for 7 days. (**A**) Representative section of laser-induced CNV lesion (labeled by yellow dashed lines) stained with hematoxylin and eosin. Scale bar: 50 μm. (**B**) Representative FFA images of the vehicle group and 0.1% FF group. (**C**) The percentage difference of CNV leakage was compared between the vehicle group (*n* = 18 animals per group) and 0.1% FF group (*n* = 12 animals per group). (**D**) Representative OCT images showing CNV (brackets). (**E**) Quantification of CNV lesion volumes in the vehicle group (*n* = 9 animals per group) and 0.1% FF group (*n* = 9 animals per group). (**F**) Representative images of flat-mounted RPE/choroid with IB4 staining (red; upper panels). Scale bar: 1000 μm. ON, optic nerve. The lower panels show higher magnification of the laser-induced CNV lesion highlighted (blue rectangle) in upper panels. Scale bar: 100 μm. (**G**) The area of CNV lesions was quantified in flat-mounted RPE/choroid with IB4 in the vehicle group (*n* = 5 animals per group) and 0.1% FF group (*n* = 5 animals per group). Data were expressed as mean ± SEM, analyzed by an unpaired Student’s *t* test.

We also evaluated the effect of the fenofibrate eye drop on laser-induced CNV. Histopathology performed on hematoxylin and eosin stains from paraffin cross-sections confirmed that there were successful laser burns with visible rupture of Bruch’s membrane in the laser-induced CNV model at 7 days after the laser photocoagulation (Figure 4A). Fundus fluorescein angiography was performed to evaluate the evolution of CNV leakage area in laser-induced CNV mice on Day 6 after laser photocoagulation (Figure 4B) in the groups with the vehicle treatment and the fenofibrate eye drop treatment. Analysis of FFAs revealed that the CNV leakage area in the fenofibrate eye drop group was 53.49% of that in the vehicle group (Figure 4C). The retinal structure and CNV volume were quantified by OCT (Figure 4D). The volume of CNV was significantly decreased in mice treated with the fenofibrate eye drop (0.008 ± 0.001 mm^3^) compared to vehicle control mice (0.020 ± 0.001 mm^3^) (Figure 4E).

Furthermore, 7 days post-CNV induction, CNV lesions were stained with IB4, and the CNV area was quantified in RPE/choroid flat-mounts (Figure 4F). Fenofibrate-treated mice showed a significantly smaller CNV area (0.039 *±*  0.010 mm^2^) than in that vehicle-treated mice (0.187  *±*  0.035 mm^2^) (Figure 4G). These results suggest that CNV was significantly suppressed by the treatment with the fenofibrate eye drop in laser-induced CNV mice.

### 3.5. Fenofibrate Eye Drop Increased PPARα Protein Levels While Attenuating Overexpression of VEGF, ICAM-1 and TNF-α in the Eyecups of Both Laser-Induced CNV Mice and the Retina of STZ-Induced Diabetic Rats

**Figure 5:**
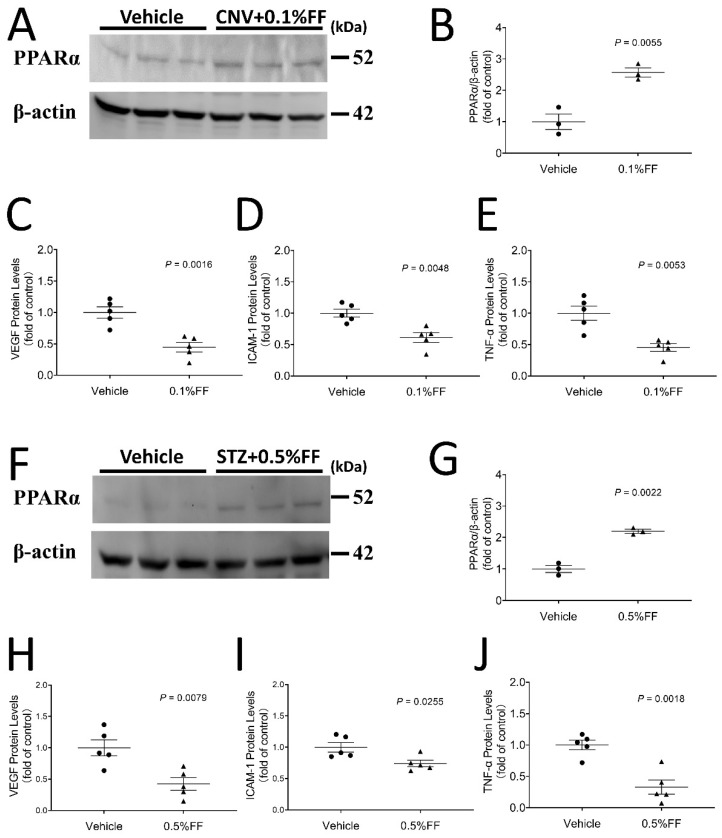
Fenofibrate eye drops increased PPARα protein expression while attenuating overexpression of VEGF, ICAM-1, and TNF-α in the eyecup of laser-induced CNV mice and in the retina of STZ-induced diabetic rat. After the eye drop administration for 7 days, the eyecup (RPE/choroid) was isolated from laser-induced CNV mice, and the retina was isolated from STZ-diabetic rats. (**A**) A representative Western blot of PPARα and β-actin in the eyecup of laser-induced CNV mice. (**B**) PPARα protein levels in the eyecup of laser-induced CNV mice were quantified by densitometry and normalized by β-actin levels (*n* = 3 animals per group). (**C**) ELISA kits were used to quantify VEGF levels in the eyecup of laser-induced CNV mice (*n* = 5 animals per group). (**D**) ICAM-1 levels in the eyecup of laser-induced CNV mice (*n* = 5 animals per group). (**E**) TNF-α levels in the eyecup of laser-induced CNV mice (*n* = 5 animals per group). (**F**) A representative Western blot of PPARα in the retina of STZ-induced diabetic rats. (**G**) PPARα protein levels in the retina of STZ-induced diabetic rats were quantified by densitometry and normalized by β-actin levels (*n* = 3 animals per group). (**H**) VEGF levels in retina of STZ-induced diabetic rats (*n* = 5 animals per group). (**I**) ICAM-1 levels in retina of STZ-induced diabetic rat (*n* = 5 animals per group). (**J**) TNF-α levels in retina of STZ-induced diabetic rats (*n* = 5 animals per group). Data were expressed as mean ± SEM, analyzed by an unpaired Student’s *t* test.

Western blot analysis demonstrated that PPARα protein levels in the RPE-choroid complex of laser-induced CNV mice (Figure 5A,B) and the retina of STZ-induced rats at 4 weeks after diabetes onset (Figure 5F,G) were significantly higher in the fenofibrate eye drop group compared with vehicle-treated animals at Day 7 of the treatment, indicating that fenofibrate activated PPARα, which upregulated its own expression in the CNV model and STZ-induced diabetic model. Full Western blots and original measurement data can be found in Supplementary Materials (Figure S1, Tables S1 and S2).

ELISA showed that inflammatory factors VEGF, ICAM-1, and TNF-α were significantly downregulated in the fenofibrate treatment group, indicating that topical administration of fenofibrate attenuated overexpression of inflammatory factors in the eyecups of the laser-induced CNV model (Figure 5C–E) and the retina of the diabetic model (Figure 5H–J).

### 3.6. Fenofibrate Eye Drops Ameliorated Retinal Inflammation and Retinal Vascular Leakage in STZ-Induced Diabetic BN Rats

**Figure 6:**
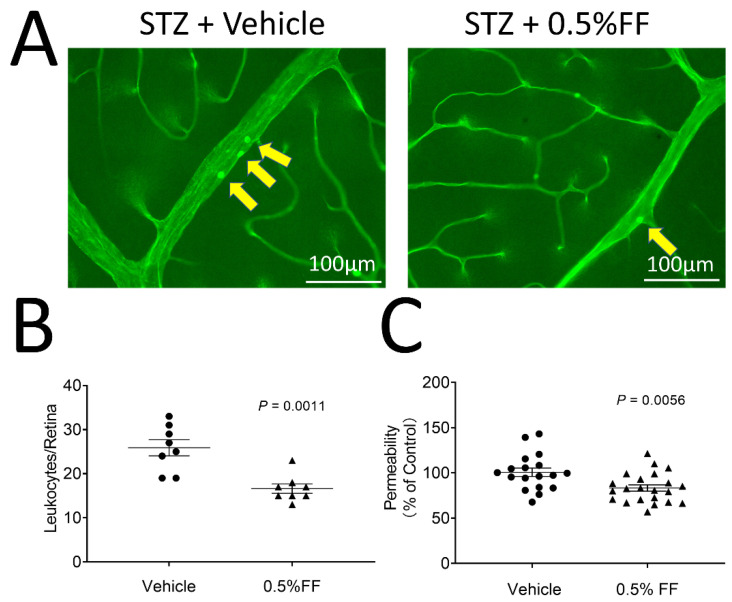
Fenofibrate eye drops ameliorated retinal vascular permeability and retinal inflammation in STZ-induced diabetes rats. Two weeks after STZ injection, diabetic rats received 0.5% fenofibrate (FF) eye drops (twice/day, 7 day). (**A**) Representative images of retinal leukostasis assay in STZ-induced diabetic rats treated with vehicle (*n* = 8 animals per group) and the FF eye drop (*n* = 8 animals per group). The arrows indicated the adherent leukocytes in retinal vessels. Scale bar: 100 μm. (**B**) Adherent leukocytes were quantified in flat-mounted retinas. (**C**) The vascular permeability was measured by spectroscopy using Evans blue dye as tracer in the vehicle group (*n* = 18 eyes per group) and 0.5% FF group (*n* = 22 eyes per group). The extracted Evans blue dye was normalized to the total retina protein concentration. Data were expressed as mean ± SEM and analyzed by an unpaired Student’s *t*-test.

We subsequently evaluated the therapeutic effect of the fenofibrate eye drop on DR in STZ-induced diabetic rats. Diabetic rats with 2 weeks of diabetes received 0.5% fenofibrate eye drops (twice/day, 7 days). As shown by the leukostasis assay, fenofibrate-treated diabetic rats showed significantly reduced adherent leukocytes in the retinal vessels compared to the vehicle-treated diabetic rats (Figure 6A,B).

We also evaluated the effects of fenofibrate eye drops on retinal vascular leakage in the diabetic rats. Fenofibrate topical application decreased retina vascular permeability by 17.36% relative to vehicle-treated STZ-diabetic rats (Figure 6C). The results suggest that the fenofibrate eye drop inhibits the retinal inflammation and reduces vascular leakage in STZ-induced diabetic rats.

### 3.7. Fenofibrate Eye Drops Had No Toxic Effects on Cornea or Retinal Structure and Function

**Figure 7:**
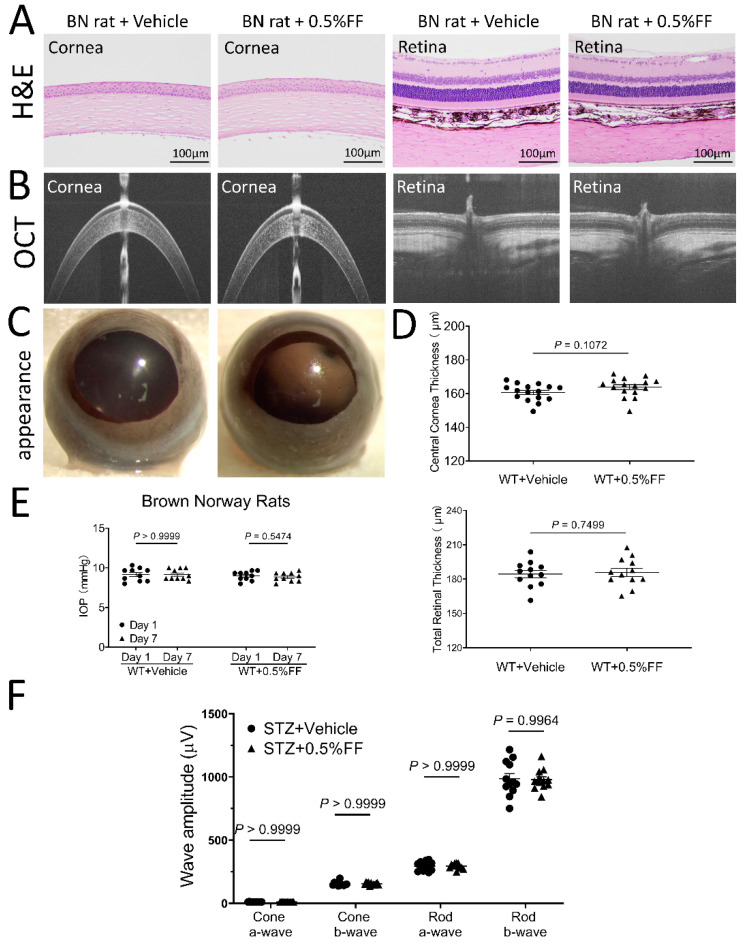
Fenofibrate eye drop had no toxic effects on cornea or retinal morphology and function in rats. (**A**) Representative H&E retinal images of the vehicle control group and 0.5% fenofibrate (FF) group. No cornea or retina structural changes were observed in the groups. Scale bar: 100 μm. (**B**) Representative OCT images of the vehicle group and 0.5% FF group. (**D**) Quantitative measures of central corneal and total retinal thickness using OCT. Values were expressed as mean ± SEM, analyzed by unpaired Student’s *t* test. (**C**) Representative images showing a perfectly clear cornea without lesion after the eye drop administration. (**E**) The measurement of intraocular pressure (IOP) after the treatment with the eye drop. (**F**) Quantification of ERG a and b wave amplitudes. Data in (**E**) and (**F**) were expressed as mean ± SEM, *n* = 12 eyes in each group, analyzed by two-way ANOVA.

**Figure 8:**
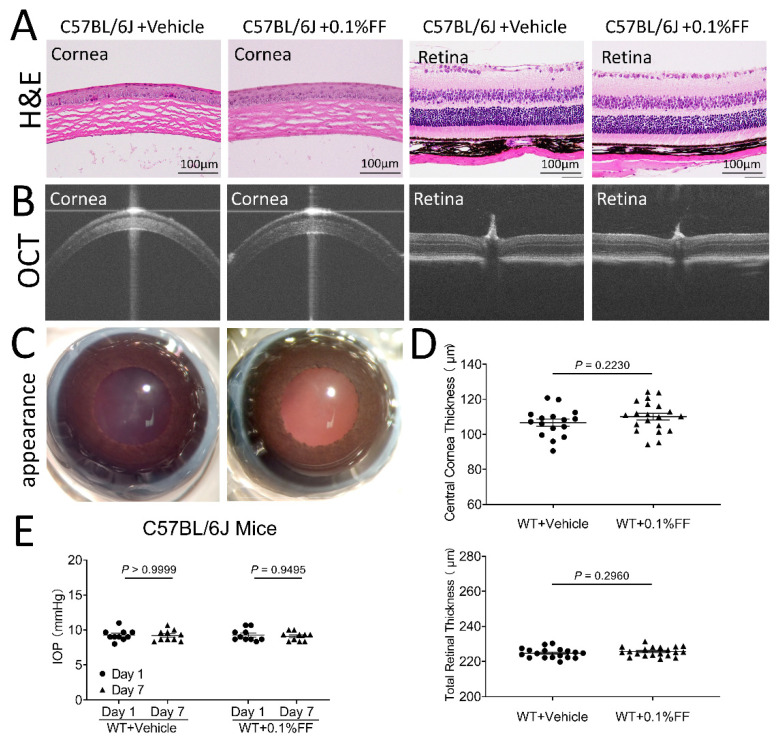
Fenofibrate eye drop had no toxic effects on cornea or retinal structure and function in mice. (**A**) Representative H&E images of the vehicle control group and the 0.1% fenofibrate (FF) group. No cornea or retina structural changes were observed. Scale bar: 100 μm. (**B**) Representative OCT images of the vehicle group and 0.1% FF group. (**C**) Representative images showing perfectly clear cornea without lesion after the eye drop administration. (**D**) Quantitative measures of central corneal and total retinal thickness using OCT. Data were expressed as mean ± SEM, *n* = 12 eyes in each group, analyzed by an unpaired Student’s *t* test. (**E**) The measurement of intraocular pressure (IOP) after the treatment with the eye drops. Data were expressed as mean ± SEM, analyzed by two-way ANOVA.

After the topical application of the fenofibrate eye drop, all animals showed clear cornea appearance, limpid anterior chamber, transparent lens, and lack of conjunctival congestion (Figure 7C and Figure 8C). Histology examination using H&E staining showed no detectable changes in the cornea or retina histology in the fenofibrate treatment group, relative to the vehicle control (Figure 7A and Figure 8A). As measured by OCT, no changes in central corneal thickness or total retinal thickness were detected in the fenofibrate-treated rats compared to the control (Figure 7B,D and Figure 8B,D). Furthermore, for functional studies, no significant differences in intraocular pressure (IOP) were observed in fenofibrate-treated diabetic rats (Figure 7E) and *Vldlr^−/−^* mice (Figure 8E). ERG was recorded to evaluate the influence of fenofibrate eye drops on retinal function. There was no significant difference in amplitudes of a or b waves of cone or rod ERG between the treatment groups and the vehicle group in rats (Figure 7F).

## 4. Discussion

Macular edema is the leading cause of vision loss in DR and neovascular AMD [1,2,23]. Retinal inflammation and subsequent BRB breakdown play a causative role in macular edema [5,33,34]. Currently, there is no non-invasive drug treatment for macular edema. In this study, we formulated fenofibrate, a PPARα agonist, into a novel, nano-emulsion-based eye drop, which delivered substantially higher amounts of the drug to the retina, compared to the systemic fenofibrate administration. Topical administration of fenofibrate eye drops conferred therapeutic effects on retinal inflammation and vascular leakage in DR and AMD models and alleviated laser-induced CNV. Further, the fenofibrate eye drop did not result in any detectable side effects to retinal function and histology. This study represents the first approach using nanoparticle-based eye drops to improve penetration of topical fenofibrate to the retina. These findings suggest that topical administration of fenofibrate eye drops has the potential to become a non-invasive therapeutic strategy for DR and AMD.

Fenofibrate is converted to its active metabolite fenofibric acid, which is a PPARα agonist. PPARα is a ligand-activated nuclear receptor that functions to control inflammatory responses by suppressing the activity of pro-inflammatory transcription factors, including nuclear factor-κB (NF-κB) and c-jun [35]. In addition, PPARα negatively regulates acute phase response (APR) signaling by reducing IL-6-induced expression of human fibrinogen genes [36,37]. Furthermore, several studies showed that PPARα agonists downregulate the biosynthesis of epoxyeicosatrienoic acids, expoxygenase products, which are pro-angiogenic. Altogether these data indicate that fenofibrate inhibits the vascular inflammatory response and NV via the PPARα pathway.

Compared to the anti-VEGF-centered treatments, accumulating evidence has indicated that fenofibrate can confer anti-angiogenic, anti-fibrotic, neuroprotective, anti-inflammatory, and anti-oxidant effects [14,15,16,17,38]. However, the therapeutic effect of systemic delivered fenofibrate is not as potent as desired, as systemic administration delivers limited amounts of the drug to the retina due to BRB, which represents a major limiting factor for improving the efficacy of fenofibrate on DR [39]. Systemic administration of fenofibrate is limited by its dose, as higher doses are associated with side effects. A recent population-based study showed a rapid raising serum creatinine level among fenofibrate users with chronic kidney disease [40]. Although the risk is low, in some cases, the therapy may increase the incidence of myopathy in diabetic patients [41]. Thus, increasing the intraocular bioavailability of fenofibrate without systemic drug overloading is critical for improving the efficacy of fenofibrate. Fenofibrate is a small molecule drug and, as such, intravitreal injection will have a short drug availability in the retina and vitreous. Our previous studies showed that PPARα agonist administration such as intravitreal injection of fenofibrate-loaded biodegradable nanoparticles effectively reduced vascular leakage and inhibited ocular NV in murine models [21]. However, IVT injection is associated with safety concerns and higher costs. Therefore, the present study attempted to develop a topic administration of fenofibrate as a noninvasive and long-term treatment of DR and AMD.

Eye drops are prescribed for long-term treatment of eye conditions. However, a major technical hurdle in topical administration of fenofibrate is its poor water solubility and ocular barriers for drug delivery to the retina. To overcome this hurdle, we designed and screened thousands of pharmaceutical formulations, from which a nano-emulsion-based formulation was identified that can deliver the drug to the retina and prolong the drug availability in the retina. In this formulation, fenofibrate in eye drops was made into a nano-emulsion. The formulation has flexible drug loading capacities for fenofibrate and is colorless and stable over six months at room temperature. The nanometer size-ranges of delivery systems offer certain distinct advantages for drug delivery [42]. In addition, nanoparticles have relatively higher intracellular uptake after delivery to tissues [43,44]. The reagents such as polysorbate added to the recipe facilitate the infiltration of fenofibrate. An early challenge faced by these technologies was their rapid clearance by the eyelids and tears. The formulation was improved by increasing the viscosity of the eye drop to keep the gel drop in the conjunctival sac for a longer time to improve the drug penetration.

As shown by pharmacokinetic studies, this nano-emulsion eye drop mediated efficient fenofibrate penetration through the ocular barriers and delivered significantly higher amounts of the drug into the retina and vitreous without overloading the drug to the liver and kidney, compared to systemic administration of a clinical dose of fenofibrate. The increased drug concentrations to the retina suggest a more potent drug effect relative to systemic administration. It is known that fenofibrate is converted to its active metabolite fenofibric acid in the plasma and tissues by esterases and becomes an active PPARα agonist. Our mass spectroscopy detected both fenofibrate and fenofibric acid in the retina and vitreous, suggesting that these tissues contain the esterases to convert fenofibrate to its active metabolite, fenofibric acid. After topical administration, the drug concentrations appeared to be a concentration gradient: sclera>choroid>retina/vitreous>lens, suggesting the drug was delivered through a periocular path and trans-sclera delivery to reach the retina and vitreous. This gradient does not seem to support that a drug delivery route of fenofibrate eye drops through penetration of the cornea, which is a very important obstacle for intraocular drug delivery. The pharmacokinetic results identified that the concentration of fenofibrate in the choroid is approximately five-fold higher than the concentration in the peripheral blood, suggesting that the drug in the posterior ocular tissues is not transported from the circulation. The trans-scleral drug delivery with periocular administration route might be a likely path mediating fenofibrate delivery to the retina.

The intact BRB is essential for preventing leakage of macromolecules and fluid from the blood vessels or choroid into the retina [45]. Increasing evidence supports that the BRB breakdown, which is regulated by multiple factors and involves different mechanisms, is a major cause of visual loss in a variety of ocular disorders [2,45,46,47]. In DR, the breakdown of BRB results in vascular leakage and subsequent DME [48]. Similarly, the alteration of the outer BRB triggers the progression to atrophic AMD [49]. Various methods have been developed for the quantitative and qualitative assessment of the BRB integrity, but each method has its particular limitations [34]. In this study, we used multiple approaches including fluorescein angiography, a permeability assay using Evans blue as the tracer, and immunocytochemical staining to evaluate the efficacy of the fenofibrate eye drop in preventing the BRB breakdown.

Our results demonstrated the effect of fenofibrate eye drops on the BRB breakdown in three distinct models: *Vldlr^−/−^* mice, the laser-induced CNV model, and the STZ-induced diabetic model. *Vldlr^−/−^* mice develop the most phenotypes of human AMD such as retinal vascular leakage, inflammation, and subretinal NV [50]. Laser-induced CNV is appealing for its ability to efficiently generate CNV lesions similar to the CNV that occurs in human AMD [50]. Previous research reported that this model of acute injury and inflammation could help to establish proof of concept for pharmacotherapy of CNV [51,52]. The STZ-induced diabetic rat model is widely used for studies of non-proliferative DR. Documented studies reported that the STZ-diabetic model develops retinal leukostasis and BRB breakdown [53]. Our previous study also showed that retinal vascular leakage occurs earlier and lasts longer in STZ-induced diabetic BN rats, suggesting that the diabetic BN rat is a good model of vascular leakage in the retina [54]. In addition, multiple studies suggested that the increase in leukocyte adhesion is a critical factor in early retinopathy or inflammatory fundus disease causing decreases in retinal blood flow and increases in inflammatory factor expression, such as vascular endothelial growth factor [28,55,56]. The present study demonstrated that the fenofibrate eye drop effectively reduced retinal vascular leakage and numbers of leukocytes in *Vldlr^−/−^* mice and STZ-diabetic BN rats, suggesting potential effects on anti-inflammation and macular edema in DR and AMD.

To establish the safety profile of the fenofibrate eye drop, we further assessed its ocular toxicities. The measurements include the clinical general apparent evaluation, measurement of IOP, ERG recording, quantification of retinal thickness using OCT, and histopathological examination. No detectable changes were observed in the retinal function and histology, suggesting topical administration of fenofibrate eye drops lacks severe toxicities at doses required for treatment.

## 5. Conclusions

In conclusion, it is necessary to explore new effective and noninvasive therapeutic modalities for macular edema in DR and AMD. The present study demonstrated that nano-emulsion-based eye drops can mediate efficient delivery of fenofibrate to the retina to reach higher concentrations than systemic administration of fenofibrate. The fenofibrate eye drop confers therapeutic effects on retinal inflammation and vascular leakage in both DR and AMD models. These findings suggest that topical application of fenofibrate or other PPARα agonists has therapeutic potential for DR and AMD.

## 6. Patents

Ronald A Wassel is an inventor of the patent covering the nano-emulsion eye drop formulation. The formulation is covered by patent number US8968775. 

## Figures and Tables

**Table 1:** The concentration of fenofibrate and fenofibric acid in various tissues of mice.

Tissue	Fenofibrate (ng/mg)		Fenofibric Acid (ng/mg)	
	IP-FF	ED-0.1%FF	IP-FF	ED-0.1%FF
Cornea	0.000 *±* 0.000	1.095 *±* 0.117 ****	0.258 *±* 0.063	7.619 *±* 1.489 **
Lens	0.000 *±* 0.000	0.501 *±* 0.032 ****	0.000 *±* 0.000	0.136 *±* 0.022 ***
Choroid	0.000 *±* 0.000	8.469 *±* 1.877 **	0.000 *±* 0.000	1.828 *±* 0.482 **
Vitreous/Retina	0.033 *±* 0.014	1.402 *±* 0.341 **	0.140 *±* 0.044	0.472 *±* 0.064 ***
Sclera	0.009 *±* 0.004	4.694 *±* 0.544 ****	0.138 *±* 0.052	2.737 *±* 0.489 **
Kidney	0.013 *±* 0.003	0.011 *±* 0.001	0.049 *±* 0.011	0.598 *±* 0.198 *
Liver	0.026 *±* 0.005	0.009 *±* 0.001 *	0.373 *±* 0.194	0.372 *±* 0.072
Plasma	1.510 *±* 0.161	1.726 *±* 0.183	86.304 *±* 22.780	157.840 *±* 62.235

IP: Intraperitoneal injection, ED: Eyedrop. mean ±SEM.; * *p* < 0.05, ** *p* < 0.01, *** *p* < 0.001, and **** *p* < 0.0001 compared with intraperitoneal injection. *n* = 5−12 eyes or organs in each group).

## Data Availability

All employed data were not published previously.

## Supplementary Materials

The following are available online at https://www.mdpi.com/article/10.3390/biology10121328/s1. Figure S1: Full Western blots, Table S1: Figure 5A Original measurement, Table S2: Figure 5F Original measurement.
