# Supplementary material for: Therapeutic Effects of Fenofibrate Nano-Emulsion Eye Drops on Retinal Vascular Leakage and Neovascularization
Source: Biology (Basel). 2021 Dec 15;10(12):1328. doi: 10.3390/biology10121328 (PMC8698460; doi:10.3390/biology10121328)
Supplement: Supplementary file 1 [file biology-10-01328-s001.zip › biology-1395345-supplementary.pdf]

Supplementary materials:

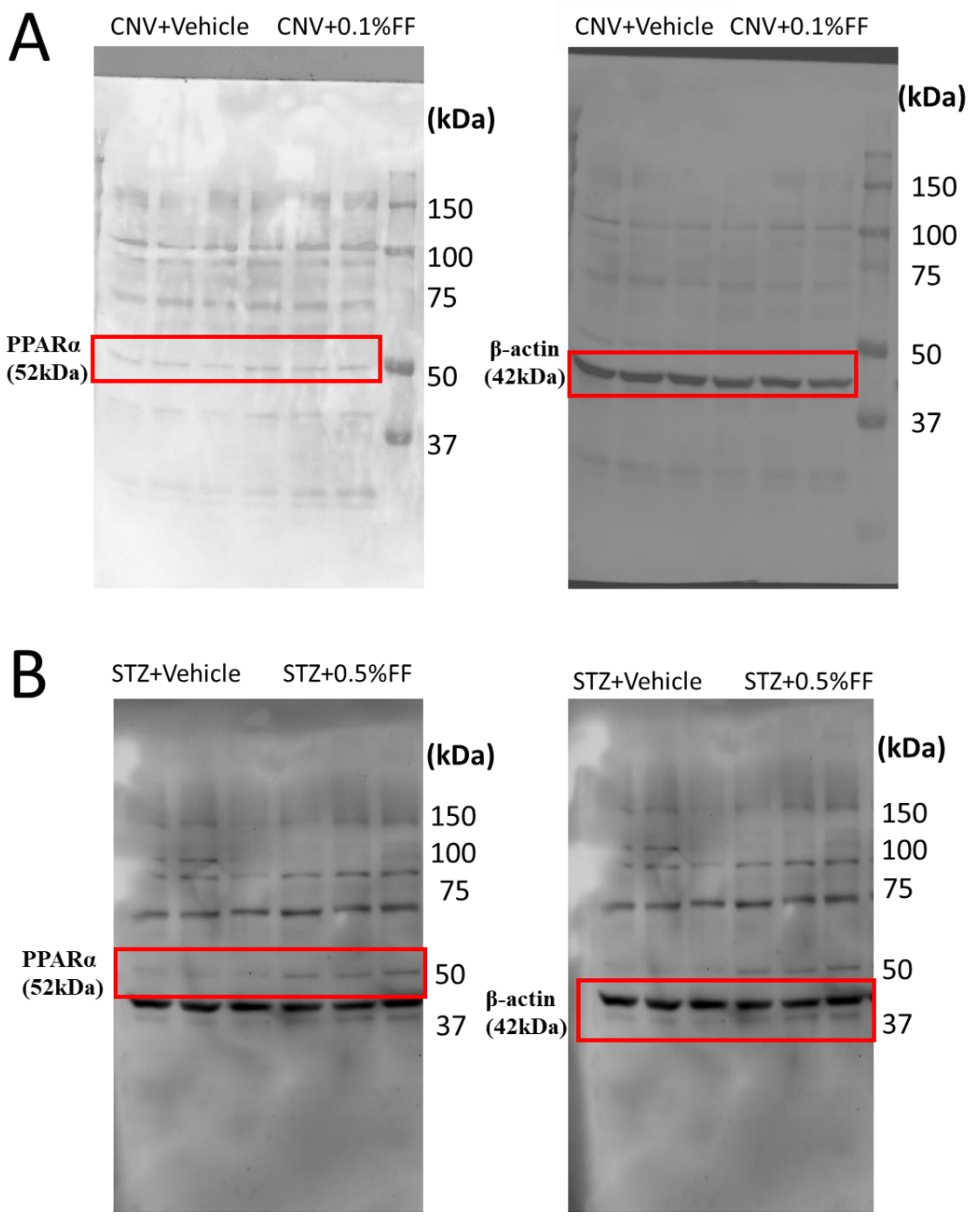

Figure S1. Full Western blots: (A) Original unedited blots indicating PPARα and β-actin for representative Western blots used in Figure 5A of the manuscript. Red rectangles are the selected for Figure 5A. The level of PPARα was analyzed with β-actin as loading control. **(B)** Original unedited blot indicating PPARα and β-actin for representative Western blots used in Figure 5F of the manuscript. Red rectangles are the selected for Figure 5F. The level of PPARα was analyzed with β-actin as loading control.

Densitometry readings/intensity ratio of each band of total blot.

Table S1: Figure 5A Original measurement

PPARα	5432.631	8783.238	5113.752	15469.95	14858.32	12188.21
β-actin	17282.22	11794.3	10774.49	10625.64	11591.18	10134.85
ratio	0.314348	0.744702	0.474617	1.455908	1.281865	1.202603

Table S2: Figure 5F Original measurement

PPARα	3964.794	4981.217	3509.51	6300.48	5545.43	5756.702
β-actin	15442.8	13140.97	10931.95	9405.702	7527.472	8247.175
ratio	0.256741	0.37906	0.321033	0.669857	0.736692	0.698021
